# Estimating intrinsic growth rates of arthropods from partial life tables using predatory mites as examples

**DOI:** 10.1007/s10493-022-00701-2

**Published:** 2022-03-14

**Authors:** Arne Janssen, Morgana Maria Fonseca, Italo Marcossi, Milena Oliveira Kalile, Andre Costa Cardoso, Adriana Helena Walerius, Aldo Hanel, Vinicius Marques, Júlia Jantsch Ferla, Vanessa Farias, Paola A. F. Carbajal, Angelo Pallini, Gösta Nachman

**Affiliations:** 1 Department of Entomology, Federal University of Viçosa, Viçosa, Minas Gerais Brazil; 2 Evolutionary and Population Biology, IBED, University of Amsterdam, Science Park 904, 1098 XH Amsterdam, The Netherlands; 3 Department of Biology, Section of Ecology and Evolution, University of Copenhagen, Copenhagen, Denmark

**Keywords:** Life table, Acari, Mesostigmata, Biological control, *r*_*m*_, Fecundity, Survival

## Abstract

The intrinsic rate of natural increase of a population (*r*_*m*_) has been in focus as a key parameter in entomology and acarology. It is considered especially important in studies of predators that are potential biological control agents of fast-growing pests such as mites, whiteflies and thrips. Life-table experiments under controlled laboratory conditions are standard procedures to estimate *r*_*m*_. However, such experiments are often time consuming and may critically depend on the precise assessment of the developmental time and the fecundity rate early in the reproductive phase. Using selected studies of predatory mites with suitable life-table data, we investigated whether and how measurements of growth rates can be simplified. We propose a new method for estimating *r*_*m*_ from partial life tables, in which the researcher can choose a level of precision based on a stand-in measure of relative error. Based on this choice, the procedure helps the researcher to decide when a life-table experiment can be terminated. Depending on the chosen precision, significant amounts of experimental time can be saved without seriously compromising the reliability of the estimated growth parameter.

**Supplementary Information:**

The online version contains supplementary material available at 10.1007/s10493-022-00701-2.

## Introduction

Life-history phenomena such as mortality and fertility patterns, as well as the age at which they occur, are crucial in understanding the population dynamics of species (Cole 1954; Caswell 2001). Population effects of stressors are increasingly studied using life tables (Forbes and Calow 2002; Stark and Banks 2003), and there is also a traditional, large and growing body of experimental work assessing the intrinsic rates of natural increase and other life-history parameters of predatory mites as natural enemies of arthropod pests. For example, a quick search in *Experimental and Applied Acarology* yielded 53 life-table studies of predatory mites, of which 62.3% mentioned biological control as ultimate target and a further 13.2% mentioned integrated pest management. Often, such studies compare various predatory mites or effects of various alternative diets on predator life histories, and they are obviously adequate for these purposes. Nevertheless, it is not clear whether complete life-table studies are needed in all cases, especially because these studies are quite time-consuming, sometimes taking more than half a year (Wen et al. 2019). Consequently, there have been several attempts trying to estimate life-history parameters based on partial life tables (Abou-Setta and Childers 1991; Stark and Banks 2016). In this paper, we review existing short-cuts for assessing intrinsic growth rates of iteroparous predatory mites and suggest a new method. The intrinsic rate of natural increase, or *r*_*m*_, is often interpreted as the maximum population growth rate under given biotic (e.g., diet) and abiotic conditions. We specifically focus on predatory mites that are intended to be used as biocontrol agents.

A typical life-history study of predatory mites starts with a cohort of eggs, which is followed throughout the juvenile and adult period, measuring the survival, development and reproduction, usually with intervals of 24 h until the last individual dies. A proper estimate of the intrinsic growth rate based on full life tables depends critically on the precise assessment of the fecundity rate and developmental time (Abou-Setta and Childers 1991). In fast-growing organisms and in organisms with low survival rates, changes in developmental rate have a larger effect on the population growth rate than increases in fecundity (Caswell and Hastings 1980). Because most predatory mites qualify as fast-growing, it is essential to accurately assess their developmental rate. For example, Van Dinh et al. (1988) showed that assessing the oviposition rate at the start of the oviposition period with intervals of 8 h is hardly enough to obtain a reliable estimate of *r*_*m*_ of two species of *Amblyseius*—and these species are not even among the phytoseiids with the highest population growth rates. So, the precision of estimates of the intrinsic growth rates of predatory mites is often negatively affected by the length of the interval between successive observations. As we show here, the precision gained by following a cohort of females until the last female dies is rather limited in comparison.

Given that the construction of life tables is often very time consuming, our aim here is to investigate whether and how procedures to assess intrinsic growth rates can be simplified, with special emphasis on biocontrol research. We do not claim that life-history studies of predatory mites have no justification in themselves, but, rather, we want to point at some screening methods that may serve as time-saving alternatives to full life-table experiments and, at the same time, still yield reasonably precise estimates of the population growth rate. For this purpose, we carried out a non-exhaustive literature search of studies of predatory mites with suitable life-table data.

### Background

A typical cohort life-table experiment is started with *N*_0_ newly laid eggs. Though the true age of these eggs may vary, often between 0 and 24 h, their age is nevertheless set to 0 days when the life table starts. The cohort is followed from day 0 until the last individual has died at age *T.* According to Carey (1993) and David et al. (1995), *r*_*m*_ can be found from the discrete version of the Lotka-Euler equation as

1 $$ \sum\limits_{x = 0}^{T} {l_{x} m_{x} e^{{ - r_{m} \left( {x + 1} \right)}} = 1,} $$

where *l*_*x*_ is the proportion of individuals still alive at age *x* and *m*_*x*_ is the average number of female eggs produced by a female of age *x*. Notice that *T* tends to increase with *N*_0_, which implies that life-table data based on small initial numbers of eggs might underestimate *r*_*m*_ compared with experiments involving large cohorts. Nevertheless, we consider the estimated values of *r*_*m*_ based on published data to be unbiased estimates of the ‘true’ *r*_*m*_, i.e., the value we would have obtained with a very large cohort. Actually, below we show that underestimating *T* often has no serious effect on the precision of *r*_*m*_.

A partial life table is defined as a life table that terminates before all individuals have died, e.g., when the individuals have reached age *a*, where *a* < *T.* In this case, we may calculate *r*_*a*_ as

2 $$ \mathop \sum \limits_{x = 0}^{a} l_{x} m_{x} e^{{ - r_{a} \left( {x + 1} \right)}} = 1, $$

where *r*_*a*_ ≤ *r*_*m*_. It may be seen that *r*_*a*_ → *r*_*m*_ as *a* → *T.*

In the following, we will consider three methods that can be used to reduce the time costs associated with life-table experiments. Each method yields an estimated value of *r*_*m*_ (denoted ${\widehat{r}}_{m})$, which is likely to differ from the value of *r*_*m*_ obtained from a full life-table experiment (i.e., if *a* = *T*). The difference between ${\widehat{r}}_{m}$ and *r*_*m*_ based on the full life table is called the relative error of ${\widehat{r}}_{m}$ and is calculated as

3 $$ RE\left( {\hat{r}_{m} } \right) = 100\frac{{\left| {r_{m} - \hat{r}_{m} } \right|}}{{r_{m} }}\% , $$

whereas the corresponding time saving in percent of the time it would take to make a full life-table experiment is found as

4 $$ S = 100\frac{T - a}{T}\% . $$

The three methods will be compared with respect to the relationship between relative error and time-saving in order to identify the method that gives the most precise estimate of *r*_*m*_ (smallest relative error) for a given value of *a.*

### Analysis of published data

Criteria for including species in our non-exhaustive review were to select phytoseiid predators that are of agricultural importance and are representative of as many families as possible (Table 1). We searched the Web of Science and Google Scholar for publications using names of genera of predatory mites that are used or considered suitable for biological control of agricultural pests. Because publications did not always present life-table data in a table, we extracted the survival and reproduction data from the figures of the publications manually or with the software Webplotdigitizer 4.0 (Rohatgi 2017). Some figures did not have sufficient resolution for this and were therefore excluded. The survey resulted in 17 papers in total (Table 1). Plots were made with extracted data and were subsequently superimposed on the published plots to ensure the adequacy of this process. We first estimated the *r*_*m*_ and the net reproductive rate (*R*_0_) of the predatory mites considering the entire life tables. After constructing a life table with all values of *x*, *l*_*x*_ and *m*_*x*_, the intrinsic growth rate can be found by numerically solving Eq. 1 or by constructing a Leslie matrix and taking the logarithm of the dominant eigenvalue.

**Table 1:** Source publications of the life-history data

Predator species	Family	Food	Temperature (°C)	References
*Amblyseius idaeus*	Phytoseiidae	*Tetranychus urticae*	25	Van Dinh et al. (1988)
*Amblyseius swirskii*	Phytoseiidae	Almond pollen	25	Riahi et al. (2017)
*Cosmolaelaps jaboticabalensis*	Laelapidae	*Protorhabditis* sp.	25	Moreira et al. (2015)
*Euseius finlandicus*	Phytoseiidae	Cherry pollen	20	Broufas and Koveos (2000)
*Euseius scutalis*	Phytoseiidae	*Panonychus citri*	25	Kasap and Şekeroğlu (2004)
*Galendromus occidentalis*	Phytoseiidae	*Tetranychus urticae*	20	Laing (1969)
*Gaeolaelaps aculeifer*	Laelapidae	*Lycoriella auripila*	23	Ajvad et al. (2018)
*Iphiseiodes zuluagai*	Phytoseiidae	*Tyrophagus putrescentiae*	25.5	Albuquerque and Moraes (2008)
*Iphiseius degenerans*	Phytoseiidae	*Tetranychus pacificus*	25	Takafuji and Chant (1976)
*Lasioseius lindquisti*	Ascidae	*Aceria dioscorides*	28	Momen et al. (2011)
*Macrocheles glaber*	Macrochelidae	*Coboldia fuscipes*	25	Wen et al. (2019)
*Neoseiulus californicus*	Phytoseiidae	*Tetranychus urticae*	25	Soltaniyan et al. (2018)
*Phytoseiulus persimilis*	Phytoseiidae	*Tetranychus urticae*	25	Bol and Janssen (unpubl)
*Proctolaelaps bickleyi*	Melicharidae	*Aceria guerreronis*	25	Lawson-Balagbo et al. (2007)
*Stratiolaelaps scimitus*	Laelapidae	*Coboldia suscipes*	25	Wen et al. (2019)
*Typhlodromalus peregrinus*	Phytoseiidae	*Tetranychus urticae*	26	Fouly et al. (1995)
*Typhlodromus pyri*	Phytoseiidae	*Eotetranychus tiliarium*	24	Kropczynska et al. (1988)

**Fig. 1:**
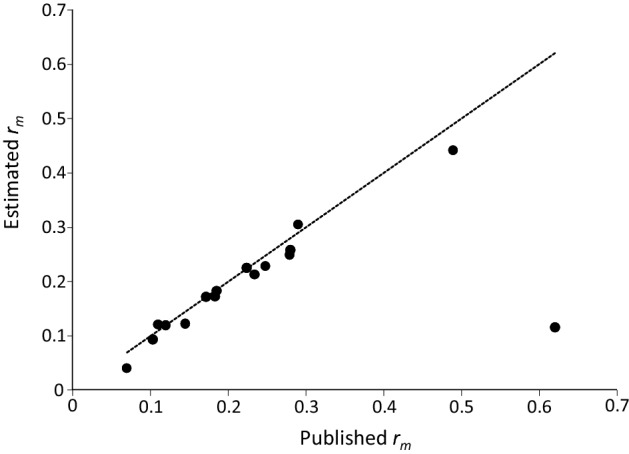
Published intrinsic growth rates (*r*_*m*_) of 17 species of predatory mites (horizontal axis) and the *r*_*m*_ we estimated using life tables extracted from these publications (vertical axis). The dashed line is that of equality (i.e., *y* = *x*). The outlier is due to a calculation error in the original paper

We compared the *r*_*m*_ values of the published data with the values estimated by us (Fig. 1). With the exception of a single paper, the published and our estimated values were reasonably similar. The exception was a study where the presented *r*_*m*_ was obviously the result of a miscalculation by the authors. We subsequently used the estimated data to demonstrate different methods to estimate intrinsic growth rates of predatory mites. The use of estimated data instead of the original data does not hamper the following analysis and conclusions in any way, because all calculations were based on the same (real or reconstructed) life tables, which are representative for a wide range of predatory mite species (Table 1).

**Fig. 2:**
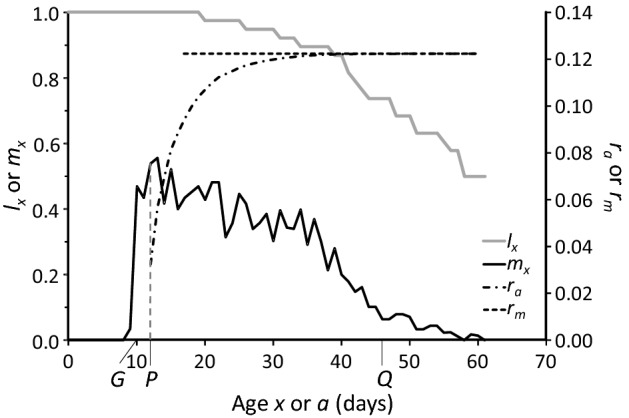
Survival and reproduction of *Euseius finlandicus* as a function of age. The solid grey line shows the survival (*l*_*x*_) until the last oviposition, the solid black line shows the production of female eggs (*m*_*x*_). The dashed-dotted line shows the value of *r*_*a*_ at age *x* = *a*, calculated from Eq. 2. The dashed horizontal line marks *r*_*m*_ based on the full life table (i.e., *r*_*m*_ = 0.1223 day^−1^). *P* (broken vertical grey line) is the age when the plateau of oviposition is reached, *G* is the generation interval (i.e., the time from egg stage to first oviposition according to Abou-Setta and Childers 1991) and *Q* is the age at 75% of the reproductive period. Notice that we give *m*_*x*_ here, which is the daily oviposition rate as given in the original publication (Broufas and Koveos 2000) multiplied by the proportion of females (0.66)

Figure 2 shows the survival (*l*_*x*_) and production of female offspring (*m*_*x*_) of the predatory mite *Euseius finlandicus* as a function of age when fed on a diet of cherry pollen at a temperature of 20 °C, with data estimated from Broufas and Koveos (2000). The development, survival and reproduction of this species is representative of phytoseiid mites, and its life table serves to explain concepts and definitions used in this study. Thus, we refer to the age at first reproduction as *x* = *G* (after Abou-Setta and Childers 1991). In the case of *E. finlandicus*, one or a few individuals started oviposition at age *x* = 9, and we assumed that the majority of the individuals started producing eggs on the next day. Hence, in general, we defined *G* as the day after the first oviposition by the cohort of individuals occurred. The age on which reproduction reaches a peak or plateau was defined as *x* = *P*. Finally, the age at reaching 75% of the reproductive period was defined as *x* = *Q* (Fig. 2).

**Fig. 3:**
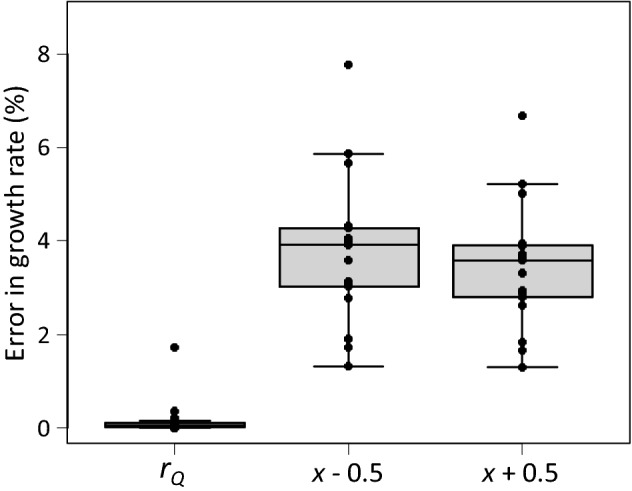
The relative error in the estimated growth rate caused by ignoring the last 25% of the reproductive period (i.e., *r*_*m*_ is estimated as *r*_*a*_ = *r*_*Q*_, where *Q* < *T*, Fig. 2), or by starting the reproductive period half a day earlier (i.e., *x’* = *x* – 0.5) or later (*x*′ = *x* + 0.5). The relative error is calculated according to Eq. 3. Shown are data points of individual species, the median (line inside boxes), 25th and 75th percentiles (boxes), and 1.5 × the interquartile range (whiskers)

The collected data span a range of population growth rates typical for predatory mites (Fig. 1). We first assessed the sensitivity of the intrinsic growth rate of all species to changes in total reproduction and in developmental rates. We removed the last 25% of the reproductive period and calculated the growth rate from age *x* = 0 to *Q* (i.e., *a* from Eq. 2 was set to *Q*, Fig. 2). The estimated values of *r*_*Q*_ were on average associated with a relative error of 0.17% compared with the values of *r*_*m*_ based on the full life tables (Fig. 3), showing that the last quarter of the reproductive period contributes marginally to the estimate of *r*_*m*_. This is because the reproductive value of a female decreases with age during the last part of the reproductive period (Carey 1993; Caswell 2001).

As explained above, observations of survival and reproduction of predatory mites are typically done once per day (see Fig. 2 for an example), indicating that the accuracy of determining the timing of first reproduction can be off with roughly half a day. To estimate the effect of this, we assumed that oviposition and survival were measured half a day earlier or half a day later (i.e., by using *x*′ = *x* ± 0.5 and calculating the intrinsic growth rate of the life table with *x*′ instead of *x*). This resulted in much larger errors in the estimated growth rate than removing the last 25% of the reproductive period (Fig. 3). It demonstrates that the precision of the intrinsic growth rate could be improved by observing more frequently, and this especially holds for the start of the oviposition period (Van Dinh et al. 1988). Hence, there is room for improvement of estimating growth rates of these phytoseiids, which, on the one hand, involves more frequent observations especially around the onset of oviposition. On the other hand, time can be saved by ignoring the last 25% of the oviposition period. Furthermore, the above analysis indicates that even more time can be saved by stopping life-table experiments earlier, without compromising the reliability of the estimated values of *r*_*m*_ significantly.

Based on these considerations, it is often not necessary to measure survival and offspring production during the entire adult life to obtain a reliable estimate of the intrinsic growth rate of predatory mite populations (Abou-Setta and Childers 1991). Once again, it should be noted that the importance of early reproduction does not hold for slow-growing organisms, for which increases in fecundity are more important than decreases in developmental rate (Caswell and Hastings 1980). Given the higher sensitivity of the intrinsic growth rates of predatory mites to the developmental rate than to the total reproduction (Fig. 3), we discuss several methods to estimate the growth rates without need to assess the full life table.

### Methods for estimating *r*_*m*_ from partial life tables

#### Estimating *r*_*m*_ for part of the reproductive period

**Fig. 4:**
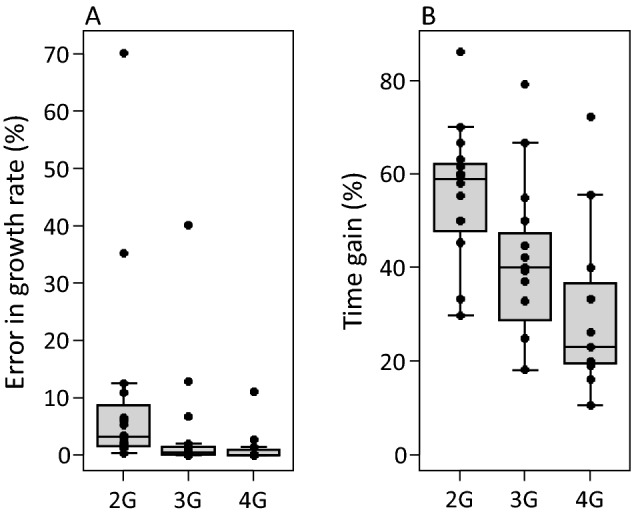
**A** Errors in the estimated growth rates (vertical axis, as in Fig. 3), based on the method by Abou-Setta and Childers (1991), calculated with a life table with a total age of 2–4 generation intervals (2–4G, Fig. 2), and **B** the time gain (in % of the total time of the full life table) resulting from this method. See Fig. 3 for explanation of the box-and-whiskers parts

Abou-Setta and Childers (1991) proposed to measure survival and oviposition of insect and mite species in time steps of a generation interval (*G*, Fig. 2). We followed the same procedure, calculating the intrinsic growth rate for a period of 2–4 × *G*. For some of the predatory mites analysed here, 3 × and 4 × G was longer than the observed total life span *T*, hence, this method could not be used for these steps. It results in considerable reduction of experimental time, especially considering a period of 2*G* (Fig. 4B), but the precision of the estimated growth rate is rather unsatisfactory (Fig. 4A).

#### Estimating *r*_*m*_ through regression with the peak oviposition

**Fig. 5:**
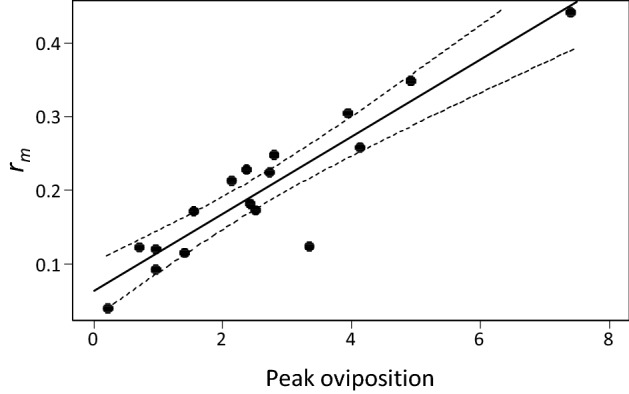
Correlation between peak rate of oviposition (*n*_*x*_ for *x* = *P*, Fig. 2) and intrinsic rate of increase (*r*_*m*_) of the 17 predatory mites; equation of the regression line (drawn): *r*_*m*_ = 0.0523*n*_*x*_ + 0.0632. The broken lines indicate the 95% confidence intervals

Janssen and Sabelis (1992) showed that the oviposition rate at the peak of oviposition (*P* in Fig. 2) correlates well with the intrinsic growth rate of predatory mites of the family Phytoseiidae that are specialized to feed on tetranychid mites. These authors suggested that the regression equation could be used to estimate the growth rate based on this peak oviposition rate. Here, we show that this correlation holds across more predatory mite families and food types (*R*^2^ = 0.84, *F*_1,15_ = 85.0, *p* < 0.001, Fig. 5), including predatory soil mites and predators of other pests such as thrips, eriophyids, flies, nematodes, as well as some that fed on pollen (Table 1). Thus, instead of conducting a full life-table experiment, the predicted intrinsic growth rate can be obtained from the straight line fitted to the *n* data points as

5 $$ \hat{r}_{m} = bm_{P} + c, $$

where *m*_*P*_ denotes the observed value of *m*_*x*_ at age *P*, whereas *b* and *c* represent the intercept and slope of the line, respectively. However, estimates of *r*_*m*_ based on the peak oviposition rates are likely to be associated with considerable uncertainty. The standard error associated with a new estimate of *r*_*m*_ can be found as (Zar 2010, Eq. 17.29):

6 $$ SE\left( {\hat{r}_{m} } \right) = \sqrt {s^{2} \left( {1 + \frac{1}{n} + \frac{{\left( {m_{P} - \overline{m}} \right)^{2} }}{{SS_{m} }}} \right)} , $$

where $\overline{m }$ is the average of the *n* peak oviposition rates used to find the straight line. *SS*_*m*_ is found as ${SS}_{m}=\sum_{i=1}^{n}{\left({m}_{i}-\overline{m }\right)}^{2}$, where *m*_*i*_ is the peak oviposition rate associated with the *i*-th species. Finally, *s*^2^ is the residual variance of the regression analysis given as

7 $$ s^{2} = \frac{1}{n - 2}\mathop \sum \limits_{i = 1}^{n} \left( {r_{i} - \hat{r}_{i} } \right)^{2} , $$

where *r*_*i*_ is the value of *r*_*m*_ obtained for the *i*-th species and ${\widehat{r}}_{i}$ is the corresponding predicted value based on Eq. 5.

As can be seen from Fig. 5, the confidence intervals around the regression line are quite wide, especially for low and high oviposition rates. This can partly be remedied by adding more data points, which will narrow the confidence limits for the predicted line (because 1/*n* will approach 0), whereas the prediction limits of *r*_*m*_ obtained from a new observation of *m*_*P*_ may still be large because they are less dependent on *n*. It should be further noticed that ordinary linear regression requires that the independent variable (i.e., *m*_*P*_) is measured without noise, and this is likely not the case. Such uncertainty in measurements of *m*_*P*_ will make the standard error of ${\widehat{r}}_{m}$ even larger.

**Fig. 6:**
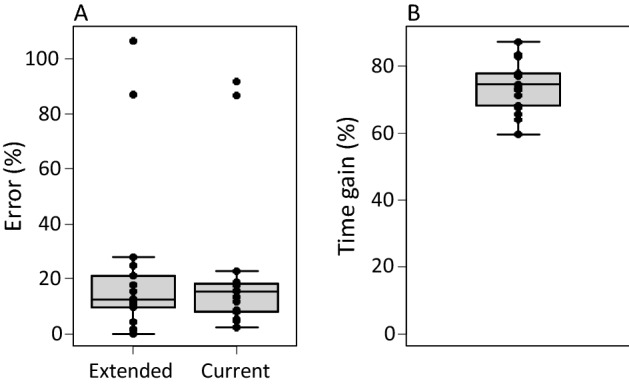
**A** Errors in the growth rates (vertical axis, as in Fig. 3) of the 17 species in this review, estimated from the regression equation of these current species (Fig. 5), and from the regression equation (*r*_*m*_ = 0.0582*n*_*x*_ + 0.0620, *R*^2^ = 0.81, *F*_1,31_ = 131.5, *p* < 0.001), fitted to the extended data set including 16 species analysed by Janssen and Sabelis (1992). **B** The time gain when using this method relative to constructing a full life table (which is equal for both estimates). See Fig. 3 for explanation of the box-and-whiskers parts

To explore the effect of sample size, we added data from Janssen and Sabelis (1992), but only of species not yet considered here. Although this narrowed the confidence intervals, there were still too few data points for high and low oviposition rates, and the error in estimating *r*_*m*_ based on the regression equation of this larger data set did not differ much from that of the dataset of the current paper (Fig. 6A). We therefore conclude that this method may serve only to obtain a rough estimate of the growth rate, especially for species with intermediate oviposition rates. Yet, the method will result in large time reductions (Fig. 6B).

#### Daily estimate *r*_*a*_ until a certain level of precision is reached

As can be seen in Fig. 2, calculating *r*_*a*_ from Eq. 2 on a daily basis yields values of *r*_*a*_ that will gradually approach *r*_*m*_ as *a* approaches *T.* This holds for all predators investigated here (Fig. S1). Such a curve can be described by an asymptotic exponential model given as:

8 $$ \hat{r}_{a} = \hat{r}_{m} \left( {1 - e^{{ - \beta \left( {a - x_{0} } \right)}} } \right), $$

where ${\widehat{r}}_{a}$ is the predicted value of *r*_*a*_ at age *a*, ${\widehat{r}}_{m}$ is the maximum value of ${\widehat{r}}_{a}$ achieved for *a* → ∞, *x*_0_ is the age for which *r*_*a*_ becomes 0, and β is a shape parameter.

From the life tables analysed here, we calculated *r*_*a*_ for each time step of 1 day, starting at age *a* = *G*. Equation 8 was then used to fit the empirical values of *r*_*a*_ using the nonlinear least squares routine nls and the package nls2 of R (Grothendieck 2013; R Core Team 2019). The model was fit to the data for all integer values of *a* > *x*_0_ + 3, because at least three positive values of *r*_*a*_ are needed to fit the three-parameter model. For each value of *a*, we obtained estimates of ${\widehat{r}}_{m}$, *x*_0_ and *β*. Based on the estimates obtained at day *a*, *r*_*m*_ can be estimated from Eq. 8 as

9 $$ \hat{r}_{m} = \frac{{r_{a} }}{{Z_{a} }}, $$

where

10 $$ Z_{a} = 1 - e^{{ - \beta \left( {a - x_{0} } \right)}} $$

is a measure of the proximity of *r*_*a*_ to ${\widehat{r}}_{m}$ if the life table is stopped at age *a*. As can be seen from the example in Fig. S2, the relative error of ${\widehat{r}}_{m}$ approaches 0 with *Z*_*a*_ approaching 1 for increasing values of *a.* In contrast to the relative error, which requires a complete life table, *Z*_*a*_ can be calculated for any given value of *a*. *Z*_*a*_ may therefore be used as a stop criterion for the duration of a life-table experiment. See Fig. S2 for a graphical explanation of the use of *Z*_*a*_ as a stop criterion.

**Fig. 7:**
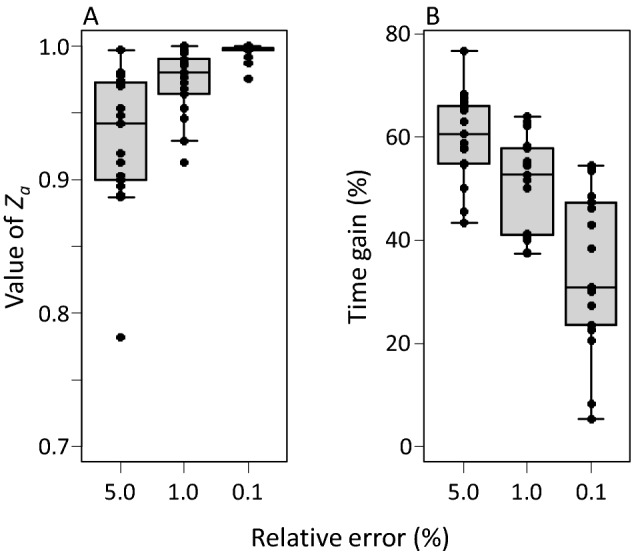
**A** The values of *Z*_*a*_ (vertical axis, Eq. 4) needed to achieve an error of 5, 1 or 0.1% in the estimated values of *r*_*m*_ compared with a full life table. **B** The time gained when using these *Z*_*a*_ as cut-off values to terminate a life-table experiment at age *x* = *a*. See Fig. 3 for explanation of the box-and-whiskers parts

We calculated the *Z*_*a*_ values for all species considered in this paper, corresponding to a relative error in ${\widehat{r}}_{m}$ equal to 5, 1 and 0.1%. The error was less than 5% for values of *Z*_*a*_ ranging from 0.782 to 0.997, whereas the error was less than 0.1% for 0.976 ≤ *Z*_*a*_ ≤ 0.99998 (Fig. 7A). The corresponding gain in experimental time was on average 60.0% for an error in ${\widehat{r}}_{m}$ <5%, and 34.3% for an error of < 0.1% (see Fig. 7B for ranges). It should be remembered that a difference in timing of the first oviposition of 0.5 day results in a change in the intrinsic growth rate of ca. 3.5% (Fig. 3), which seems to be acceptable for most researchers. Hence, depending on the precision desired, a significant proportion of experimental time can be gained by using this method.

**Fig. 8:**
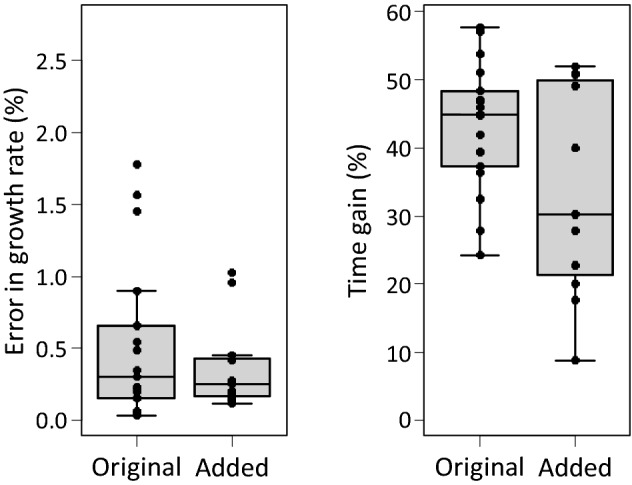
**A** Errors in the growth rates (vertical axis, as in Fig. 3) of the 17 species included in this review (Original) and with 11 other life tables obtained at various temperatures (Added), using a fixed cut-off value of *Z*_*a*_ (Eq. 10) of 0.99. **B** The time gained when *Z*_*a*_ is set to 0.99. See Fig. 3 for explanation of the box-and-whiskers parts

To test the generality of the proposed method, we supplemented the original data set composed of 17 predatory mite species with a new data set of 11 life tables obtained at various temperatures (Table S1) and analysed both data sets using a cut-off value of *Z*_*x*_ = 0.99. This resulted in an average error in the growth rate of 0.53% and a time gain of 43.4% for the original data, whereas the error and time gain were 0.38% and 33.6%, respectively, for the new data set (Fig. 8).

In contrast to the two other estimation methods described above, the third method requires some cumbersome calculations. One of us (GN) has therefore developed a user-friendly program called *Life Table Assistant*, which can be used to enter daily (or hourly) observations on survival (*l*_*x*_) and reproduction (*m*_*x*_). The latter is the average number of female eggs, so the numbers of eggs need to be corrected for the sex ratio. We suggest that the sex ratio can be obtained by rearing a cohort of eggs to adulthood, without any need for following the fate of each individual separately on a daily basis. For each new observation at day *a*, *l*_*a*_ and *m*_*a*_ are entered, the program updates the model parameters and provides the current values of *r*_0_, *Z*_*a*_ and ${\widehat{r}}_{m}$. Values are also shown as graphs, making the estimation process more transparent. [The program is developed in Delphi XE8 (Embarkcadero®), runs under Microsoft Windows, and can soon be obtained freely in the near future by sending an email to gnachman@bio.ku.dk].

## Discussion

We evaluate two existing methods, and propose a third-new-method to estimate the intrinsic rate of natural increase of predatory mites. All three methods save considerable time compared to carrying out a full life-table analysis. It should be noted that the methods specifically apply to predatory mites, but probably also to organisms with similar life histories (e.g., spider mites and insects with comparable generation times). The first method reviewed is that of Abou-Setta and Childers (1991), and its precision is not impressive. The second method is based on an earlier publication of one of us (Janssen and Sabelis 1992), and in retrospect, this method also does not offer the precision often required, especially not for species with high or low oviposition rates. The third method is probably the most general of the three methods, as it is basically the same as the standard method used for studying life-histories, but applies a stop criterion as a guideline for when a life-table experiment can be terminated. This criterion can be chosen according to a desired level of precision. The method allows saving significant amounts of experimental time without losing much precision in the estimate of the intrinsic growth rate (Fig. 8). A method along similar lines was proposed by Stark and Banks (2016), but their stop criterion is based on the estimates of life-history parameters obtained from partial life tables being not significantly different from that of the full life table. It is not only difficult to assess this criterion without conducting a full life-table experiment, it is also problematic to define a cut-off point by means of statistical inference, because a value that is not significantly different does not guarantee that the estimate is precise. In fact, the relative error associated with the estimated values of *r*_*m*_ varied from ca. 9 to 26% (Stark and Banks 2016), which is considerably higher than the errors obtained here (Fig. 8).

The intrinsic growth rates obtained with the full life-table analysis used for predatory mites may seem to be precise, but our analysis indicates that they are likely to be biased because they are sensitive to the timing of first reproduction, especially in species with relatively high population growth rates. Therefore, if the aim is to obtain accurate estimates of the intrinsic growth rate using a full life table, it is recommended to record the first few days of the oviposition period with intervals of 12 h for species with growth rates comparable to *E. scutalis* and *A. idaeus*, and with intervals of 6 h for faster growing species such as *P. persimilis* and *P. bickleyi*. For slower species, such as *M. glaber* and *S. scimitus*, intervals of 24 h are probably sufficient. Still, many life tables of predatory mites are based on daily observations, but fortunately, there is growing awareness of the importance of assessing the onset or reproduction more accurately (e.g., Uddin et al. 2017; Azevedo et al. 2018).

Other life-history parameters such as the net reproduction (*R*_0_) and the generation time (*T*_*c*_) are less sensitive to the exact onset of reproduction, but they will also asymptotically approach their final value as cohort age approaches maximum age (*T*) in a similar manner as the intrinsic growth rate (Fig. S3). This suggests that these parameters can be estimated with a procedure similar to that used to estimate *r*_*m*_ described under method 3.

The importance of the intrinsic growth rate of predatory mites for biocontrol purposes can be questioned. It is certainly true that several pests are better controlled through the augmentative release of predatory mites with a high intrinsic growth rate, for example, the control of *Tetranychus urticae* with *P. persimilis*. However, augmentative control by slower growing predatory mites can be achieved through releasing higher numbers of predators (Janssen and Sabelis 1992). Additionally, populations of natural enemies can be maintained in a crop through provision of alternative food, which allows them to suppress the growth of small, colonizing populations of pests (Huffaker and Kennett 1956; de Klerk and Ramakers 1986; van Rijn et al. 1999, 2002). In such cases, the intrinsic growth rates of these predators are less important than their total reproduction.

If measuring the growth rate is part of an assessment of the suitability of the predator as biocontrol agent, an alternative approach would be to estimate the instantaneous population growth rate during a population dynamics experiment. This is especially practical if such an experiment is needed for testing the biocontrol capacities of a predatory mite. For pests of plants, for example, such an experiment would be done on single plants inhabited by pest and the potential biocontrol agent. Given that the predator population, released after the prey has been established on the host plant, will initially not be limited by food supply, it will start growing exponentially and this exponential phase of the dynamics can be used to estimate its growth rate by fitting the equation

$$ N_{t} = N_{0} e^{rt} $$

to repeated observations of *N*_*t*_. Though estimated values of *r*_*m*_ obtained from intact plants may reflect more realistic environmental conditions than those provided during life-table experiments (Walthall and Stark 1997; Sibly 1999; Nachman and Zemek 2003; Poletti and Omoto 2012; Rezende et al. 2013; Lima et al. 2016), the former method is likely to be associated with considerable bias due to sampling error, age- and stage-structure, variable climatic conditions, food quality, etc.

In general, we do not advocate against conducting complete life-table studies because such studies may be essential, for example, to detect effects of toxicants on population growth rates, where it is unclear which life-history variables and life stages are most sensitive (Forbes and Calow 2002; Stark and Banks 2003). For semelparous predators, it is even inevitable to construct a complete life table (Muñoz-Cárdenas et al. 2014). Nevertheless, we propose that full life-table studies can be replaced by the partial life-table studies for several purposes, resulting in reasonably precise estimates of the intrinsic growth rate. For example, of the 53 life-table studies of predatory mites found in *Experimental and Applied Acarology*, only three did not use the full life table to estimate the population growth rate. The other publications contained a total of 225 different estimates of intrinsic growth rates for which the method proposed here could be applied, resulting in considerable savings of precious experimental time. Furthermore, we suggest that especially the third method based on partial life tables can be used for other taxonomic groups than predatory mites, such as arthropods with similar reproduction biologies (e.g., phytophagous mites and insects).

## Supplementary Information

Below is the link to the electronic supplementary material.

Supplementary file1 (DOCX 212 kb)

## Data Availability

Life-table data will be made available upon acceptance (10.21942/uva.19242873).

## References

[CR1] Abou-Setta M, Childers C (1991). Intrinsic rate of increase over different generation time intervals of insect and mite species with overlapping generations. Ann Entomol Soc Am.

[CR2] Ajvad FT, Madadi H, Michaud J (2018). Life table of *Gaeolaelaps aculeifer* (Acari: Laelapidae) feeding on larvae of *Lycoriella auripila* (Diptera: Sciaridae) with stage-specific estimates of consumption. Biocontrol Sci Technol.

[CR3] Azevedo LH, Ferreira MP, de Campos CR (2018). Potential of *Macrocheles* species (Acari: Mesostigmata: Macrochelidae) as control agents of harmful flies (Diptera) and biology of *Macrocheles embersoni* Azevedo, Castilho and Berto on *Stomoxys calcitrans* (L.) and *Musca domestica* L. (Diptera: Muscidae). Biol Control.

[CR4] Broufas G, Koveos D (2000). Effect of different pollens on development, survivorship and reproduction of *Euseius finlandicus* (Acari: Phytoseiidae). Environ Entomol.

[CR5] Carey JR (1993). Applied demography for biologists, with special emphasis on insects.

[CR6] Caswell H (2001). Matrix population models. Construction, analysis, and interpretation.

[CR7] Caswell H, Hastings A (1980). Fecundity, developmental time, and population growth rate: an analytical solution. Theor Popul Biol.

[CR8] Cole LC (1954). The population consequences of life history phenomena. Q Rev Biol.

[CR9] David J-F, Celerier M-L, Henry C (1995). Note on the use of the basic equation of demography. Oikos.

[CR10] de Albuquerque FA, de Moraes GJ (2008). Perspectivas para a criação massal de *Iphiseiodes zuluagai* Denmark & Muma (Acari: Phytoseiidae). Neotrop Entomol.

[CR11] de Klerk M, Ramakers P (1986). Monitoring population densities of the phytoseiid predator *Amblyseius cucumeris* and its prey after large scale introductions to control *Thrips tabaci* on sweet pepper. Meded Fac Landbouwwet Rijksuniv Gent.

[CR12] Forbes VE, Calow P (2002). Population growth rate as a basis for ecological risk assessment of toxic chemicals. Philos Trans R Soc Lond B Biol Sci.

[CR13] Fouly AH, Abou-Setta MM, Childers CC (1995). Effects of diet on the biology and life tables of *Typhlodromalus peregrinus* (Acari: Phytoseiidae). Environ Entomol.

[CR14] Grothendieck G (2013) nls2: non-linear regression with brute force

[CR15] Huffaker CB, Kennett C (1956). Experimental studies on predation: predation and cyclamen-mite populations on strawberries in California. Hilgardia.

[CR16] Janssen A, Sabelis MW (1992). Phytoseiid life-histories, local predator–prey dynamics, and strategies for control of tetranychid mites. Exp Appl Acarol.

[CR17] Kasap I, Sekeroglu E (2004). Life history of *Euseius scutalis* feeding on citrus red mite *Panonychus citri* at various temperatures. Biocontrol.

[CR18] Kropczynska D, Van de Vrie M, Tomczyk A (1988). Bionomics of *Eotetranychus tiliarium* and its phytoseiid predators. Exp Appl Acarol.

[CR19] Laing J (1969). Life history and life table of *Metaseiulus occidentalis*. Ann Entomol Soc Am.

[CR20] Lawson-Balagbo LM, Gondim MGC, de Moraes GJ (2007). Life history of the predatory mites *Neoseiulus paspalivorus* and *Proctolaelaps bickleyi*, candidates for biological control of *Aceria guerreronis*. Exp Appl Acarol.

[CR21] Lima DB, Melo JWS, Gondim MGC (2016). Population-level effects of abamectin, azadirachtin and fenpyroximate on the predatory mite *Neoseiulus baraki*. Exp Appl Acarol.

[CR23] Momen F, Abou-Elela M, Metwally A (2011). Biology and feeding habits of the predacious mite, *Lasioseius lindquisti* (Acari: Ascidae) from Egypt. Acta Phytopathol Entomol Hung.

[CR24] Moreira GF, de Morais MR, Busoli AC, de Moraes GJ (2015). Life cycle of *Cosmolaelaps jaboticabalensis* (Acari: Mesostigmata: Laelapidae) on *Frankliniella occidentalis* (Thysanoptera: Thripidae) and two factitious food sources. Exp Appl Acarol.

[CR25] Muñoz-Cárdenas K, Fuentes LS, Cantor RF (2014). Generalist red velvet mite predator (*Balaustium* sp.) performs better on a mixed diet. Exp Appl Acarol.

[CR26] Nachman G, Zemek R (2003). Interactions in a tritrophic acarine predator–prey metapopulation system V: Within-plant dynamics of *Phytoseiulus persimilis* and *Tetranychus urticae* (Acari: Phytoseiidae, Tetranychidae). Exp Appl Acarol.

[CR27] Poletti M, Omoto C (2012). Susceptibility to deltamethrin in the predatory mites *Neoseiulus californicus* and *Phytoseiulus macropilis* (Acari: Phytoseiidae) populations in protected ornamental crops in Brazil. Exp Appl Acarol.

[CR28] R Core Team (2019) R: A language and environment for statistical computing. Version 3.6.0. R Foundation for Statistical Computing, Vienna, Austria. http://www.R-project.org

[CR29] Rezende DDM, Fadini MAM, Oliveira HG (2013). Fitness costs associated with low-level dimethoate resistance in *Phytoseiulus macropilis*. Exp Appl Acarol.

[CR30] Riahi E, Fathipour Y, Talebi AA, Mehrabadi M (2017). Natural diets versus factitious prey: comparative effects on development, fecundity and life table of *Amblyseius swirskii* (Acari: Phytoseiidae). Syst Appl Acarol.

[CR31] Rohatgi A (2017) WebPlotDigitizer. Version 4.0. Austin, Texas, USA. https://apps.automeris.io/wpd/

[CR32] Sibly RM (1999). Efficient experimental designs for studying stress and population density in animal populations. Ecol Appl.

[CR33] Soltaniyan A, Kheradmand K, Fathipour Y, Shirdel D (2018). Suitability of pollen from different plant species as alternative food sources for *Neoseiulus californicus* (Acari: Phytoseiidae) in comparison with a natural prey. J Econ Entomol.

[CR34] Stark JD, Banks JE (2003). Population-level effects of pesticides and other toxicants on arthropods. Annu Rev Entomol.

[CR35] Stark JD, Banks JE (2016). Developing demographic toxicity data: optimizing effort for predicting population outcomes. PeerJ.

[CR36] Takafuji A, Chant DA (1976). Comparative studies of two species of predaceous phytoseiid mites with special reference to the density of their prey. Res Popul Ecol.

[CR38] Uddin MN, Alam MZ, Miah MRU (2017). Life table parameters of an indigenous strain of *Neoseiulus californicus* McGregor (Acari: Phytoseiidae) when fed *Tetranychus urticae* Koch (Acari: Tetranychidae). Entomol Res.

[CR39] Van Dinh N, Janssen A, Sabelis MW (1988). Reproductive success of *Amblyseius idaeus* and *Amblyseius anonymus* on a diet of two-spotted spider mites. Exp Appl Acarol.

[CR40] van Rijn PCJ, van Houten YM, Sabelis MW (1999). Pollen improves thrips control with predatory mites. Bull IOBCWPRS.

[CR41] van Rijn PCJ, van Houten YM, Sabelis MW (2002). How plants benefit from providing food to predators even when it is also edible to herbivores. Ecology.

[CR42] Walthall WK, Stark JD (1997). Comparison of two population-level ecotoxicological endpoints: the intrinsic (r_m_) and instantaneous (r_i_) rates of increase. Environ Toxicol Chem Int J.

[CR43] Wen M-F, Chi H, Lian Y-X (2019). Population characteristics of *Macrocheles glaber* (Acari: Macrochelidae) and *Stratiolaelaps scimitus* (Acari: Laelapidae) reared on a mushroom fly *Coboldia fuscipes* (Diptera: Scatopsidae). Insect Sci.

[CR44] Zar JH (2010). Biostatistical analysis.

