# Supplementary material for: Estimating intrinsic growth rates of arthropods from partial life tables using predatory mites as examples
Source: Exp Appl Acarol. 2022 Mar 14;86(3):327–42. doi: 10.1007/s10493-022-00701-2 (PMC8967767; doi:10.1007/s10493-022-00701-2)
Supplement: Supplementary file 1 — Supplementary file1 (DOCX 212 kb) [file 10493_2022_701_MOESM1_ESM.docx]

**Supporting Figures and Table.**

**Figure S1.** The values of *r_a_* as a function of the last age (*x* = *a*) included in the partial life tables used to calculate the growth rate (Equation 2). *r_a_* is seen to approach an asymptote, which coincides with the intrinsic growth rate (*r_m_*) of the species based on the analysis of the full life table. Each curve represents one of the species reviewed here.

**Figure S2.** The value of *Z_a_* (Equation 10; black curve, left vertical axis) and the estimated growth rate ($\hat{r}_{m}$, Equation 9; green curve, right vertical axis) as a function of the last age (*a*) included in the partial life tables for the species *Euseius finlandicus* (see Fig. 2). The value of *r_m_* obtained from the full life table (i.e., *r_m_* = 0.1223 day^-1^) is shown here as a reference (broken black line). *Z_a_* approaches a value of 1 when $\hat{r}_{m}$ approaches *r_m_*. The cut-off procedure for estimating the growth rate can be visualised as follows. The broken red line extending from the left vertical axis indicates a *Z_a_* value of 0.95; the 1st day on which this value is surpassed is on day 24 (vertical broken red line). The $\hat{r}_{m}$ value obtained for this day is 0.1198 (horizontal broken line extending towards the right vertical axis), with a relative error of 2.03% (notice the scale of the right-hand axis). The broken blue lines show that a value of *Z_a_* > 0.99 is reached on day 31, with $\hat{r}_{m}$ = 0.1214 and a relative error of 0.66%; the broken dark green lines correspond to *Z_a_* > 0.999, day 43, with $\hat{r}_{m}$ = 0.1223 and a relative error of 0.03%.

**Figure S3.** The estimated values of net reproduction *R*_0_ (**A**) and the generation time (**B**) as function of the last age (*a*) included in the partial life tables used to calculate these two life-history parameters. Right-hand points of the curves are the ‘true’ *R_0_* and *T_c_*, estimated with the full life table (*a* = *T*). Each curve represents one of the species reviewed here. The curves level of towards the end of the curve, but for later ages than those of the intrinsic growth rate (Fig. S1). See legend to Fig. S1 for further explanation.

**Table S1.** Source publications of further life-history data.

| **Predator species** | **Family** | **Food** | **Temperature** | **References** |
| --- | --- | --- | --- | --- |
| *Euseius scutalis* | Phytoseiidae | *Panonychus citri* | 20, 39 | Kasap & Sekeroglu (2004) |
| *Gaeolaelaps aculeifer* | Laelapidae | *Tyrophagus putrescentiae* | 18.5, 22.5, 24.5, 27.5 | Lobbes & Schotten (1980) |
| *Iphiseius degenerans* | Phytoseiidae | *Tetranychus urticae* | 15, 30 | Tsoukanas et al. (2006) |
| *Neoseiulus californicus* | Phytoseiidae | *Tetranychus urticae* | 20, 30 | Uddin et al. (2017) |
| *Typhlodromus pyri* | Phytoseiidae | *Eotetranychus tiliarium* | 12–20 | Kropczynska et al. (1988) |
